# Mass Cytometry Imaging for the Study of Human Diseases—Applications and Data Analysis Strategies

**DOI:** 10.3389/fimmu.2019.02657

**Published:** 2019-11-14

**Authors:** Heeva Baharlou, Nicolas P. Canete, Anthony L. Cunningham, Andrew N. Harman, Ellis Patrick

**Affiliations:** ^1^The Westmead Institute for Medical Research, University of Sydney, Westmead, NSW, Australia; ^2^Centre for Virus Research, The Westmead Institute for Medical Research, Westmead, NSW, Australia; ^3^School of Mathematics and Statistics, University of Sydney, Sydney, NSW, Australia

**Keywords:** imaging cytometry, analysis, multiplexed ion beam imaging, imaging mass cytometry (IMC), mass cytometry (CyTOF), cytometry, multiplexed imaging, single cell

## Abstract

High parameter imaging is an important tool in the life sciences for both discovery and healthcare applications. Imaging Mass Cytometry (IMC) and Multiplexed Ion Beam Imaging (MIBI) are two relatively recent technologies which enable clinical samples to be simultaneously analyzed for up to 40 parameters at subcellular resolution. Importantly, these “Mass Cytometry Imaging” (MCI) modalities are being rapidly adopted for studies of the immune system in both health and disease. In this review we discuss, first, the various applications of MCI to date. Second, due to the inherent challenge of analyzing high parameter spatial data, we discuss the various approaches that have been employed for the processing and analysis of data from MCI experiments.

## Introduction

Multiplexed imaging methods are becoming an increasingly important tool for both basic science and clinical research (1–10). Recently, mass cytometry imaging (MCI) approaches, which enable imaging at subcellular resolution have been described (11, 12). MCI enables up to 40 parameters to be visualized in a single tissue section and is being rapidly adopted for various applications, including studies in cancer, diabetes and the definition of complex immune subsets during development and homeostasis (13–21).

### Mass Cytometry Imaging Technologies

**Figure 1:**
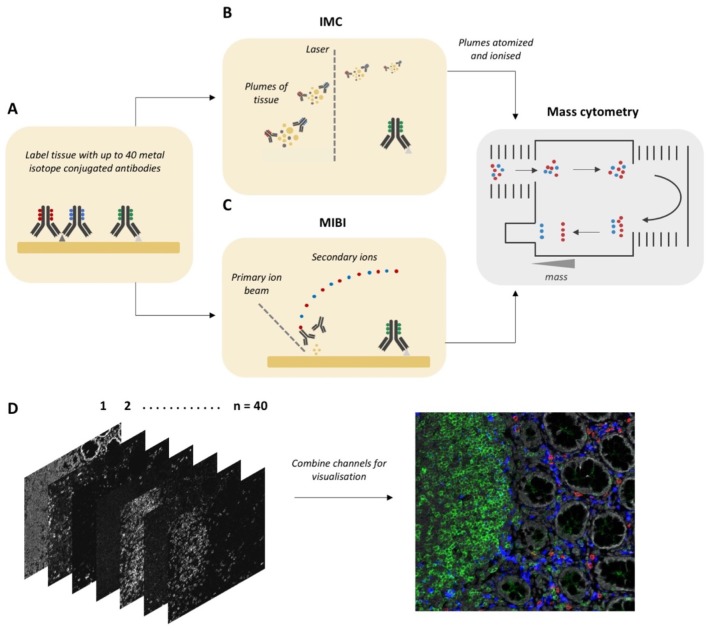
Workflow for Mass Cytometry Imaging. **(A)** Tissue sections are first labeled with a cocktail of metal-isotope-tagged antibodies. **(B)** In Imaging Mass Cytometry the tissue is ablated using a laser with 1 μm spot size. Plumes of tissue matter are then aerosolized, atomized and ionized, and then fed into a time-of-flight mass spectrometer, where metal ions are separated based on mass. **(C)** In Multiplexed Ion Beam Imaging a thin layer of the sample surface is ablated using an oxygen-based primary ion beam. Metal isotypes are liberated from antibodies as secondary ions which are then delivered to a time-of-flight mass spectrometer. **(D)** A high dimensional image is generated, which when combined and visualized, resembles a traditional fluorescence microscopy image. Parts of this figure were made Biorender.

There are two approaches for MCI—Imaging Mass Cytometry (IMC) (11) and Multiplexed Ion Beam Imaging (MIBI) (12). In both methods, the first step is the labeling of tissue sections with up to 40 different antibodies conjugated to stable isotopes, mostly from the lanthanide series (Figure 1A). In IMC, the tissue is then ablated using a laser with a 1 μm spot size, which rasterizes over a selected region of interest. Plumes of tissue matter are then aerosolized, atomized, and ionized, and then fed into a time-of-flight mass spectrometer for analysis of isotope abundance (Figure 1B). In MIBI, an oxygen duoplasmatron primary ion beam rasterizes over the tissue, ablating a thin layer of the tissue surface, which then liberates antibody-bound metal isotopes as ions. Similar to IMC, these secondary ions are then fed into a time-of-flight mass spectrometer for the estimation of isotope abundance [Figure 1C; (13)]. In both methods, the isotope abundance of each “spot” can then be mapped back to the original co-ordinates, producing a high dimensional image qualitatively similar to a fluorescence microscopy image (Figure 1D).

The differences between IMC and MIBI have previously been reviewed (2). However, MIBI has undergone extensive improvements since its initial description, overcoming many of the limitations relating to speed of acquisition and multiplexing capacity (13). Accordingly, there are no up-to-date published comparisons of these two technologies. Both have recently been used to successfully analyse 30–40 parameters *in situ* in patient tissue samples (13, 17, 18). Two important differences we will mention relate to sample ablation and image resolution. IMC uses a laser for sample acquisition and is designed to ablate the entire sample with a fixed lateral resolution of 1,000 nm. However, MIBI utilizes a tuneable ion beam which can be adjusted for varying depth of sample acquisition and also ion spot size (image resolution). This means that the same area can be scanned at a lower resolution to gain an overview and then potential areas of interest rescanned at a higher resolution, reportedly as low as 260 nm, though with a trade-off of longer acquisition times. A comparison of features between IMC and MIBI is summarized in Table 1.

**Table 1:** Highly multiplexed imaging technologies.

	**Serial staining immunofluorescence**	**Metal tagged antibodies**	
Examples	CycIF, GEMultiOmyx, 4i, CODEX	IMC	MIBI
Resolution	~200 nm	~1,000 nm	~260 nm^*^
Simultaneous detection limit	1–5	40	40
Max number of epitopes imaged per section^**^	~60	40	40
Throughput^***^	Hours or 1 day per cycle per tissue section	1 mm^2^/2 h	1 mm^2^/5 h (500 nm resolution)
References	(3, 4, 6–8)	(11)	(12, 13)

* *A smaller spot size (resolution) results in longer acquisition times. A lower limit of 260 nm is referenced in a recent publication, but the actual data acquired in the study was at a resolution of 500 nm (13)*.

** *There is no hard upper limit for serial staining protocols, but published data has shown approximately 60 markers per section (4, 7, 8). A limit of 40 markers for IMC and MIBI is derived from interpretation, based on both the indicated references and current reagent availability*.

*** *The rate-limiting step for serial staining protocols is the antibody incubation period which can take hours and is often performed overnight. Throughput for IMC is listed in the Fluidigm product specification sheet for acquisition at 200 Hz. Throughput for MIBI is based on correspondence with IONpath and is expected to be published later this year in a paper describing the current specifications of MIBI*.

### Significance of Mass Cytometry Imaging

MCI is a landmark development because it allows for upward of 40 markers to be simultaneously stained, acquired and visualized, enabling a variety of distinct cell types to be analyzed concurrently in their native microenvironment. The microenvironment consists of a complex matrix of fluids, proteins and cells which provide signals that shape a given cells phenotype and function within an organ in both health and disease (22–26). Indeed, there is increasing evidence that cellular functions are programmed not just by cell ontogeny but also by signals from the surrounding microenvironment. Examples include Monocytes and Dendritic Cells and T cells which exist in several functionally diverse subsets, which vary across tissues so as to meet the requirements of their local environment (27–31). Specific subsets of Dendritic Cells, Innate Lymphoid Cells and T cells can carry out distinct functions at a given point in time, inducing either tolerance or inflammation depending on a host of signals derived from both cytokines and direct cell contact (32–35). In the context of disease pathogenesis, the tumor microenvironment is now appreciated as a complex signaling network between transformed and non-transformed cells, with the latter being corrupted to promote tumor function (36, 37). The importance of the microenvironment for cell function is clear. The major contribution of MCI is that it provides spatial data for a large number of parameters at subcellular resolution. As such, we are now positioned to discover interdependencies between complex cell subsets in health and disease. These interactions can be further investigated *ex vivo* to determine their functional outcome and contribution to disease progression.

MCI is also an important development for practical reasons as it enables complete studies to be performed on archival samples. This is particularly useful as research questions evolve with time and it is invaluable to be able to repeatedly interrogate the same sample for different parameters. This feature will be particularly helpful for investigations of inflammatory disorders where significant heterogeneity can exist, making it difficult to accurately characterize the cell types involved and thus the immune motifs underlying the disease; such is the case for dendritic cell subsets which are partly defined by surface markers that are labile during inflammation (38). Furthermore, many studies can only be performed using small biopsies or precious post-mortem samples, as in brain and pancreatic tissues, with samples typically curated through biobank networks (39, 40). As such large gaps remain in our understanding of disease pathogenesis in these tissues; a gap which MCI is poised to fill.

### Other Approaches for Highly Multiplexed Imaging

#### Serial Staining Immunofluorescence

Other approaches exist which are fluorescence-based and involve iterative rounds of staining, imaging, and removal of fluorescent signals (3, 4, 6–9). In these serial staining approaches, typically 2–3 parameters are acquired per round, thus requiring 13–20 rounds to acquire 40 parameters which is the current limit for MCI. Advantages of this approach relate to broad compatibility with many fluorescence-based imaging systems and the capacity to acquire large areas across multiple tissue sections in a short period of time, which allows parallel processing of many slides. However, there are several disadvantages including lengthy acquisition times which can span weeks, extensive tissue manipulation and perturbance of antigens between staining cycles, autofluorescence, and the lower dynamic range of fluorescence compared to MCI (3, 8, 41, 42). Further, considerable expertise and computing power is required to process the resultant large images, which if acquired at a high resolution in multiple Z planes, can form gigabytes and even terabytes of raw data, which must be deconvolved, projected and registered prior to analysis. For basic science research, our evaluation is that these methods could complement each other; where MCI captures a global overview and serial staining immunofluorescence could be used to quickly answer targeted questions with fewer parameters, using a large cohort of samples. However, in the clinical setting, a serial staining method that relies on chemically induced signal removal is unlikely to be adopted, as there will always be questions relating to incomplete signal removal and also antigenic stability over time. A comparison of features between serial staining and MCI methods is provided in Table 1.

#### Mass Spectrometry Imaging

It is worth noting that MCI differs significantly from other Mass Spectrometry Imaging (MSI) approaches such as Matrix Assisted Laser Desorption/Ionization (MALDI) MSI. In MALDI-MSI, a laser and mass spectrometer are used to ablate and ionize molecules on the surface of a sample and the mass spectrum of each pixel on the section is collected. This is performed in a label-free manner, whereby the identity of molecules, such as proteins and metabolites, is determined either by fragmentation of ionized species at each pixel, or by comparing the intact mass to a database of known molecules (43–45). In this way, MALDI-MSI has much greater coverage compared to MCI techniques. However, MALDI-MSI has several limitations compared to MCI, such as lower resolution, lower sensitivity (often limiting analysis to larger proteins) and compatibility issues with common sample preservation methods such as formalin fixation or embedding in optimal cutting temperature compound (OCT) (46–49). The MSI community is currently at work to address these limitations and this has recently been reviewed (46). In particular, once limitations in resolution and sample preparation requirements are bridged, this could offer exciting opportunities for multi-modal imaging protocols which combine the breadth of MSI with the sensitivity of MCI, allowing for in-depth molecular profiling of targeted cell subsets.

The purpose of this review is two-fold. First, we provide an overview of the published applications of MCI. Second, as analysis is a significant challenge in MCI projects, we provide an overview and evaluation of the data processing and analysis strategies that have been successfully employed. The reader will take away an understanding of the applications and questions that can be answered with MCI, as well as the various possible approaches for analysis to address these.

## Applications of Mass Cytometry Imaging

**Figure 2:**
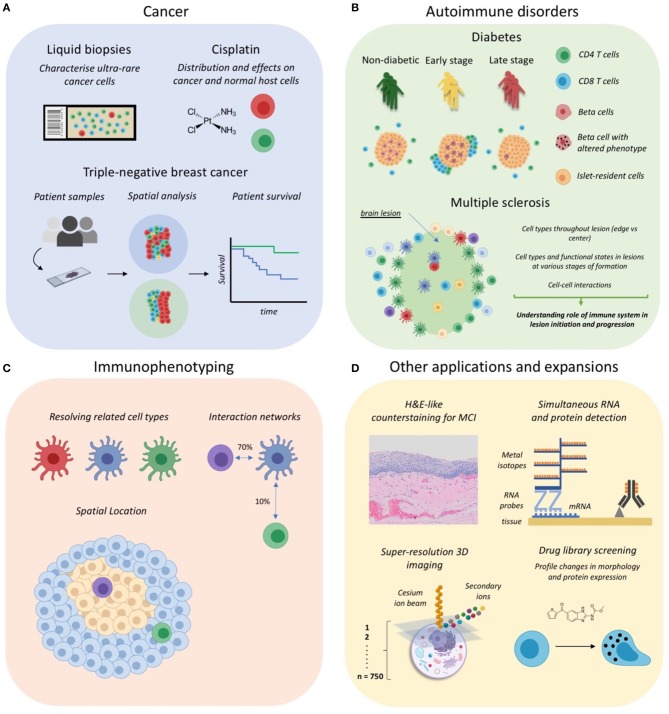
Applications of Mass Cytometry Imaging. **(A)** MCI has been utilized for cancer studies examining rare circulating tumor cells in liquid biopsies, the distribution and effects of anti-cancer drugs such as cisplatin, and for profiling the tumor-immune landscape in tripe-negative breast cancer. **(B)** IMC has been used to investigate the immune correlates of autoimmune disorder progression, including type 1 diabetes mellitus and multiple sclerosis lesion formation in the central nervous system. **(C)** Some studies have begun to use MCI for immunophenotyping so as to discriminate cell subsets, their interactions and anatomical distribution. **(D)** Several recent expansions of MCI, including the development of a counterstaining method, simultaneous RNA and protein detection, 3D super-resolution imaging of single cells, and applications for drug screening. Parts of this figure were made Biorender.

In this section we will discuss all applications of MCI to date which are summarized in Table 2 with associated references and also graphically represented in Figure 2. MCI has been primarily used in the fields of cancer research and more recently in studies of autoimmune disorders such as type 1 diabetes mellitus and multiple sclerosis. MCI has also been used for immunophenotyping studies to define complex cell types, their interactions and also location *in situ*. Additionally, there have been many recent expansions, which include a counterstaining method, RNA detection, drug discovery, and also 3D imaging. For each study we will refer to the specific technology applied—IMC or MIBI—and also to the application of these technologies generally, as MCI.

**Table 2:** Summary of MCI applications and associated publications.

**Application**	**Description of applications**	**References**
Original papers	Landmark papers describing IMC and MIBI	(11, 12)
Cancer	Phenotyping cancer cells in Liquid Biopsies & tissue touch preparations	(14, 15)
	Distribution and cellular effects of platinum-based drug Cisplatin	(50, 51)
	Analyzing tumor-microenvironment to predict patient outcomes	(13, 52, 53)
Autoimmune disorders	Immune system involvement in type 1 diabetes progression	(17, 18)
	Profiling immune cells in lesions at different stages of lesion progression	(54)
	Landscape of microglia and astrocytes in MS lesions	(55)
Immunophenotyping	Resolving phenotype, location and function of murine kidney myeloid subsets	(19)
	Demonstration of interactions between antigen presenting cells and memory T cells in the fetal small intestine	(20)
	Mapping location myeloid subsets in human tonsil tissue	(56)
	Mapping location of memory and marginal zone B cells in human appendiceal tissue	(21)
Other applications and expansions	Counterstaining method for IMC, generating H&E like image.	(57)
	Simultaneous detection of RNA transcripts and protein by IMC.	(16)
	3D super-resolution MIBI	(51, 58)
	High content drug screening	(59)

### Cancer

The first published examples using primary cancer tissues were the original papers describing IMC and MIBI (11, 12). These were more proof of principle studies largely confirming pathologist observations and findings from the literature. Subsequent studies in cancer research adapted IMC for the analysis of very small clinical samples (14, 15) (Figure 2A). Gerdtsson et al. showed that IMC can be effectively integrated into the established high definition single cell analysis (HD-SCA) assay for liquid biopsies. HD-SCA involves coating glass slides with millions of cells from a blood draw and using immunofluorescence to identify and characterize very rare tumor cells among millions of leukocytes (60). The major advantages of performing microscopy in this context, is that it allows small samples to be analyzed and is an almost lossless strategy capturing cells that might otherwise be missed using suspension cytometry (60). The HD-SCA assay has been shown to generate useful data for clinical decision making (61). Gerdtsson et al. used a combination approach where immunofluorescence identified regions with tumor cells, which were then acquired by IMC for in-depth phenotyping. This approach was later used by the same group on touch preparations of bone marrow and prostate tissue from a patient with polymetastatic prostate cancer (14). A key finding from their IMC analysis was the lower expression of EpCAM on tumor cells in the bone marrow compared to the prostate. This would suggest that for the patient under study, anti-EpCAM therapies may have limited effect on metastatic tumor cells in the bone marrow. Therefore, these studies highlight the utility of MCI to examine multiple clinically relevant markers in small samples, which can be used to guide therapeutic interventions. The identification of biomarkers of metastases is an active field of research and combined genomics and MCI data would be a powerful approach for discovery analysis in limited patient samples (62, 63).

IMC has also been used to investigate the spatial distribution, and effect on relevant cell populations, of platinum-based drugs *in situ*, which are used to treat solid tumors (50). In this study the drug cisplatin was detected by its atomic mass of 195, in a patient-derived pancreatic ductal adenocarcinoma xenograft model (Figure 2A). Traditionally, cisplatin distribution has been measured using inductively coupled plasma mass spectrometry which does not allow cellular resolution (64, 65). However, the use of IMC has overcome this limitation, successfully showing differential cisplatin entry into tissue compartments as well as cell-type dependent effects on DNA damage and proliferation (50). Furthermore, a recent pre-print from the Nolan lab has described an expansion of MIBI capable of super-resolution 3D imaging (discussed further below) (51). This technology was used to map the intracellular distribution of cisplatin in an ovarian cancer cell line. Together, these MCI modalities offer complementary approaches to studying the effects of cisplatin on both tumor and normal host cells. This will be particularly useful for the development of therapeutic interventions targeting the kidney and cochlea tissues where cisplatin accumulation often leads to loss of kidney function (66) and hearing, respectively (67, 68).

Recent studies have used MCI to analyse the tumor microenvironment for patterns of prognostic value. The first such study was performed by Keren et al., who published a landmark paper using MIBI to profile the tumor immune landscape in archival samples from 41 triple-negative breast cancer patients (13). Importantly, this study was the first to use MCI as a standalone technique for the comprehensive profiling of immune cells and their spatial orientation *in situ*. As such, this study provides a framework for the analysis of MCI data which is discussed throughout the section below on “Image Processing and Analysis.” Through the innovative use of spatial analysis techniques, the authors found that patients could be stratified into two groups defined as either “mixed,” with extensive tumor and immune cell mixing, or “compartmentalized,” in which regions comprised mostly of either tumor or immune cells (Figure 2A). This stratification turned out to be meaningful and was predictive of many differences between patients, including immune cell composition, immunoregulatory protein expression and even patient survival, where “compartmentalized” patients were significantly more likely to survive compared to their “mixed” counterparts (Figure 2A). Importantly, this example demonstrates how sophisticated computational tools can be used to model high dimensional spatial data, revealing immune network patterns which are predictive of disease outcome.

Another recent study by Carvajal-Hausdorf et al. used an IMC panel to investigate the tissue microenvironment in breast cancer patients and risk of relapse following trastuzumab (HER2 extracellular domain-targeted) treatment (52). They showed that the ratio of the HER2 extracellular/intracellular domain expression correlated with benefit from trastuzumab treatment, and were able to relate this to CD8 T cell proximity to tumor cells. Worth noting, the authors mention that they were previously unable to generate a reliable predictive ratio using fluorescence microscopy due to difficulty normalizing for variable quantum yield between fluorophores, an issue overcome through the use of IMC, again highlighting its potential for clinical applications.

In all, MCI is an emerging and powerful tool for applications in clinical management, and also preclinical studies examining drug effects and the tumor microenvironment.

### Autoimmune Disorders

#### Diabetes

Two papers recently published back-to-back in Cell Metabolism, have set the scene for the use of IMC to investigate the immune correlates of type 1 diabetes mellitus (T1DM) progression (17, 18). These studies compared the pancreas of healthy organ donors to that of T1DM patients at various times since diagnosis (Figure 2B). To date human T1DM studies have been limited by both sample availability and the availability of highly multiplexed imaging methods to comprehensively analyse these archival tissue samples. Here we highlight a few key results from these studies which used IMC as a standalone tool for their investigations.

A hallmark of T1DM is the progressive loss of beta cells in the islets of the pancreas and we are only beginning to understand the role the immune system plays in this loss which includes the generation of islet-reactive CD8 T cells (69, 70) and how their function is promoted by (71, 72) or inhibited (73) by other resident immune cell subsets. In their study, Damond et al. developed a 35 parameter panel to investigate the correlates of beta cell loss. They showed that at the time of T1DM onset, beta cell levels are similar to that of healthy pancreata, but that the expression of beta-cell markers varies widely across islets of the same donor, with only some resembling that of late stage disease (>8 years) (Figure 2B). In essence, this revealed that at the time of initial diagnosis beta cells, which normally produce insulin, are present but look different; thus, highlighting the possibility of therapeutic interventions to rescue beta cells during early stage disease.

Both studies found a temporal correlation between beta cell destruction and CD4/CD8 T cell infiltration into the islets (Figure 2B). Considering the coincident downregulation of beta cell markers, it has been hypothesized that this may be a form of “immunological camouflage” in an attempt to escape immune attack (74). Furthermore, alpha cells, which are also resident within islets, were shown to upregulate the beta-cell transcription factor NKX6, which supports the idea of alpha cell trans-differentiation into beta cells in the case of beta cell loss. Together these studies illustrate the enormous utility of MCI for understanding the causes of T1DM and for developing new strategies for prevention and cure.

#### Multiple Sclerosis

Multiple sclerosis is a progressive disease characterized by the appearance of demyelinating lesions in the central nervous system (75). The formation of these lesions occurs in stages which have been formally characterized (76). Importantly, lesion formation is considered an immune-mediated pathology. However, similar to T1DM, dissecting the role of the immune system has been challenging, due to both difficulty obtaining samples and the lack of technologies to simultaneously characterize the repertoire, functional state and spatial location of lesion-associated immune cell populations (77–79). Accordingly, two recent pre-prints by Ramaglia et al. and Park et al. have utilized IMC to characterize the immune environment of post-mortem MS brain tissue which we discuss in this section (54, 55).

The study by Ramaglia et al. was specifically purposed as a proof of principle as to the utility of IMC to profile the immune landscape of MS lesions (54). Accordingly, their results largely recapitulate previous literature, but importantly demonstrate that a wealth of information from previous studies can be captured in a single section. One specific example is the finding that the majority of demyelinating macrophages are derived from the resident microglial pool and not activated blood-derived macrophages. Such information may be useful to guide therapy design, as it suggests existing therapies, which block the influx of inflammatory cells, may need to be complemented with therapies targeting the factors that drive microglia activation (80–82).

Park et al. used a panel of 13 markers to perform a targeted examination of myeloid and astrocyte phenotypes in MS lesions (55). A key strength of this study is the employment of sophisticated computational tools which demonstrate both diversity and specific ordering of immune networks within MS lesions. They were able to identify five subtypes of astrocytes and six subtypes of myeloid cells within MS lesions, the latter of which were shown to localize to different areas of the lesion, suggesting distinct functional states. Further they were able to show there were significant cell-cell interactions between specific immune subsets in hypercellular regions of lesions indicating that cell to cell communication within the lesion is ordered and not random. However, the limited parameters in this study did not allow for the assessment of the functional consequence of these interactions. Furthermore, they were able to quantify the influence of cell intrinsic and extrinsic factors on cellular marker expression. This showed that microglia on the edge of the lesion are more responsive to cues from the extracellular environment, whilst microglia within the lesion are more driven by cell intrinsic programs potentially instigated by myelin phagocytosis.

Together, these studies provide a strong rationale for follow-up MCI studies with increased parameters and patient samples to further characterize the immune networks that are active in various stages of lesion development. The use of complementary technologies such as laser-capture microdissection and RNA-sequencing may aid in this characterization through the selective capture and in-depth profiling of selected areas of interest (83).

### Immunophenotyping of Cell Subsets

The definition of distinct immune cell subsets is an important area of research for the study of human diseases. For example, cells that are ontogenically and therefore phenotypically closely related, can have distinct immunological functions and consequently play very different roles in disease pathogenesis (38, 84). Furthermore, these many and varied subsets will have their own specific interaction networks, which adds another layer of complexity. Accordingly, some studies have begun to use MCI for the definition of complex cell subsets, their anatomical location and potential interacting neighbors (Figure 2C).

The deep phenotyping enabled by MCI enables phenotypically similar cell types to be distinguished. This was demonstrated by Brahler et al. who used IMC to define Dendritic Cell and Macrophage subsets in the murine kidney. In this work, contrary to previous studies, it was shown that CD11c-expressing Macrophages and not Dendritic Cells form a dense dendritic network throughout the kidney (19). Additionally, they defined two Dendritic Cell subsets, characterized as expressing either CD103 or CD11b and localizing to large and small blood vessels, respectively. Follow-up depletion studies showed that in models of kidney inflammation CD11b+ Dendritic Cells played a pro-inflammatory role, whilst CD103+ Dendritic Cells were regulatory in nature. Importantly, previous studies which depleted CD11c-expressing Dendritic Cells showed attenuation of local inflammatory responses in mouse models of inflammation (85, 86). The high parameter characterization enabled by IMC demonstrated that among Dendritic Cells, it was likely the depletion of the CD11b-expressing Dendritic Cell subset that led to mitigation of local inflammation.

In its early adoption IMC has proven a useful tool for making qualitative observations of the location of multiple cell subsets and their interactions. In particular it has been used to show clustering of activated memory CD4+ T cells with specific antigen presenting cells in the fetal small intestine, potentially indicative of immune priming by antigen presenting cells (20). This helped cement the results from their *ex vivo* data which demonstrated a diverse and active memory T cell compartment within the fetal small intestine, a site previously considered to be protected from foreign antigens (87). Another study by Durand et al. investigated the role of myeloid cell subsets in CD4+ T follicular helper (Tfh) cell priming, which is critical for the generation of effective humoral responses (56). IMC analysis was used to map the anatomical location of myeloid cell subsets in human tonsil tissue. Critically, Macrophages were shown to cluster with Tfh cells which supported *ex vivo* data showing that Macrophages are potent inducers of Tfh cells. Finally, a study by Zhao et al. investigated the relationship between memory B cells and their marginal zone counterparts which are related but reported to play distinct roles in memory responses and the generation of innate responses independent of T cell antigens, respectively (21, 88, 89). In their study, IMC revealed that class switched memory B cells (CD27+IgM-) are located toward the periphery of appendiceal lymphoid tissue, closer to the follicle associated epithelium, and surround their marginal zone B cell (CD27+IgM+IgD+) counterparts. The differential location supports the notion of differing functional roles of these subsets, however the significance of the differential localization observed here remains to be elucidated (19, 20).

With increased adoption, MCI will likely become an indispensable tool for atlas studies of immune cell composition, interactions, and anatomical location in health and disease.

### Other Applications and Expansions

The first adaptation of IMC was its aforementioned use for phenotyping liquid biopsies from cancer patients (14, 15). Beyond this, several techniques have been developed which either add to, or extend on, IMC and MIBI (Figure 2D).

In histology, counterstaining, most commonly using haematoxylin, is useful as it provides an overview of tissue architecture and can assist in the assessment of tissue pathology. Accordingly, Catena et al. developed a counterstaining method for IMC using ruthenium tetraoxide, which achieves good uniformity, paralleling that of a haemotoxylin stain (57). In combination with the DNA intercalator, iridium, the output images, when pseudo-colored appropriately, are similar to that of a traditional H&E stain (Figure 2D). Importantly, this method was shown to not interfere with detection of signals due to metal-tagged antibodies. Furthermore, H&E images have been shown to provide sufficient information for Dermatologist-level classification of skin cancer using deep neural networks (90). Accordingly, the counterstaining method described here could provide useful textual information for automated classification of anatomically distinct structures.

IMC has been used for the detection of proteins and compounds with appropriate atomic mass. However, Schulz et al. have described an extension of IMC which includes the detection of RNA transcripts *in situ* (16). This entailed a modification of the popular RNAscope technique where the final amplification steps use lanthanide-tagged, rather than enzyme-tagged, oligonucleotides [Figure 2D; (91)]. The authors show a good correlation of both protein and RNA signals between the modified and usual fluorescence-based version of the RNAscope assay. However, they note a lower limit of detection of 6–14 transcripts per cell, which is significantly lower than the single transcript sensitivity of the original assay. This lower sensitivity is likely due to the loss of enzyme-substrate amplification present in the original assay. This could potentially be improved through the use of lanthanide-tagged substrates, or using lanthanide-tagged antibodies targeting the deposited substrate.

The capacity to detect RNA transcripts by IMC represents a significant extension of this technique. For example, many pathogens, such as HIV, cannot reliably be detected *in situ* using antibodies and require RNA-based detection (92). This opens the door to the investigation of host-pathogen interactions in a high parameter setting. In addition, difficult to target or lowly expressed proteins, such as cytokines, can now be detected *in situ*, and mapped to the cell types producing these functional molecules (93–95). Indeed, this was demonstrated by Schulz et al., where they assessed CXCL10 expression in breast cancer tissues. They found that CXCL10-expressing cells clustered together and that their frequency correlated with T cell presence. CXCL10 expression has been associated with poor survival in various cancers (96–98). As such, further profiling of the specific T cell subsets recruited, their phenotype and localization in the tumor microenvironment, could help explain the oncogenic effects of CXCL10 in driving metastasis and poor clinical outcome.

Two recent pre-prints from the Nolan lab have described an extension of MIBI which allows three-dimensional imaging at sub 100 nm resolution (51, 58). This technique involves taking multiple axial scans of single cells using a cesium ion beam, which can then be reconstructed to form a 3D image with lateral (XY) and axial (Z) resolutions of approximately 30 and 5 nm, respectively (Figure 2D). One drawback of this approach is that the cesium beam cannot efficiently ionize lanthanide-tagged antibodies. Accordingly, the authors developed a novel antibody tagging method, where stable isotopes (for example ^19^F, ^81^Br, ^127^I) are embedded into single-stranded DNA oligonucleotide-tagged antibodies, which can be efficiently ionized by the cesium ion beam. The inutility of lanthanides suggests a trade-off of resolution for multiplexing capacity. This form of “super-resolution” MIBI is designed for the analysis of single cells rather than large fields of view containing thousands of cells, as in IMC and MIBI. Accordingly, the types of questions which can be asked and answered differ vastly. As such, we will not further discuss the use of “super resolution” MIBI for remainder of this review.

Finally, a recent preprint by Bouzekri et al. has described a workflow for the use of IMC for high content drug screening [Figure 2D; (59)]. A key challenge of these screens is the labor and cost of testing thousands of compounds across many cell lines. However, it has been shown that high-dimensional profiling of drug responses in a single cell line can help select a subset of compounds with diverse biological performance, which by definition is a good library for screening drug effects (99). Accordingly, as the speed and resolution of IMC improves, it could become a useful tool for the improved screening of preclinical drug candidates (100).

### Summary of MCI Applications

Despite its recency, MCI has already proven a useful tool for various applications, particularly in the domain of clinical and translational research (Figure 2, top panel). As outlined here, several recent studies have begun to use MCI as a primary research tool for the systems level interrogation of patient samples (13, 16–18). Accordingly, a variety of techniques for image processing and analysis were employed to identify changes in cell composition, phenotype and spatial organization, which we have comprehensively reviewed here. A common feature of these studies was the use of panels to define canonical cell types (Macrophages, T cells, B cells etc.) along with a selection of disease-relevant markers such beta cell markers in the diabetes studies (17, 18) and immunoregulatory protein markers in the breast-cancer cohort study (13). This provided a valuable overview of the distinct lineages that may play a role in disease pathogenesis and is therefore a good approach for pilot studies seeking to understanding the role of the immune system in disease etiology. Going forward, however, we anticipate studies that will become more tailored, examining specific lineages and functional markers which are known players in a specific disease context. Indeed, as discussed here, data from the few comprehensive MCI studies to date have provided a rationale for the detailed examination of specific subsets of T cells (16–18) in the patient samples studied. In the context of translational research, these targeted investigations are necessary as interventions that enhance or inhibit the activity of specific cell types require their precise definition (53, 101–106).

Finally, it is important to discuss the potential of MCI in deciphering the nature of unknown targets. This relates to the definition of “novel cell subsets” and “novel phenotypes,” respectively. In terms of cell subsets, with the large number of parameters offered by MCI, it may be tempting to characterize potential “novel” cell subsets. However, caution should be exercised as cell segmentation will always be imperfect, potentially leading to the erroneous classification of new cell types. In saying that, information gleaned from MCI may provide a useful hint at phenotypes which could be validated using more robust single-cell techniques such as flow or mass cytometry (15). MCI will likely be more useful in defining the relative spatial distribution of cell subsets classified using unbiased approaches such as single-cell RNA-seq (107). Regarding novel phenotypes, disease often accompanies phenotypic changes in known target cells, which has been demonstrated in several MCI papers discussed in this review (13, 16–18). Markers of known relevance to each disease were studied, however it is possible for MCI to be combined with other multi-omics tools in donor matched samples to screen for differentially expressed molecules in health and disease (101, 108, 109). This form of guided panel design would potentiate novel discoveries through the mapping of functionally relevant markers to specific target cells or spatial niches.

## Image Processing and Analysis

Due to its ease of use, MCI is poised to be a useful tool in clinical research. However, a key bottleneck in MCI is related to both image processing and the inherent difficulty of analyzing up to 40 parameters with added spatial dimensions. In this section, we first cover all techniques that have been used for processing MCI data. Broadly, this includes image denoising, single-cell segmentation and finally tissue and cell-type annotation. Next, we discuss approaches for the analysis of MCI images that have been implemented in MCI studies at present for studying disease models. The analysis section is formatted as a series of general biological questions which can be answered using image analysis. For each question we discuss both its clinical significance and the specific techniques used in MCI studies to answer each question.

### Processing

In this section, we outline the steps taken to process MCI images, allowing downstream analysis with a single-cell approach.

#### Denoising

**Figure 3:**
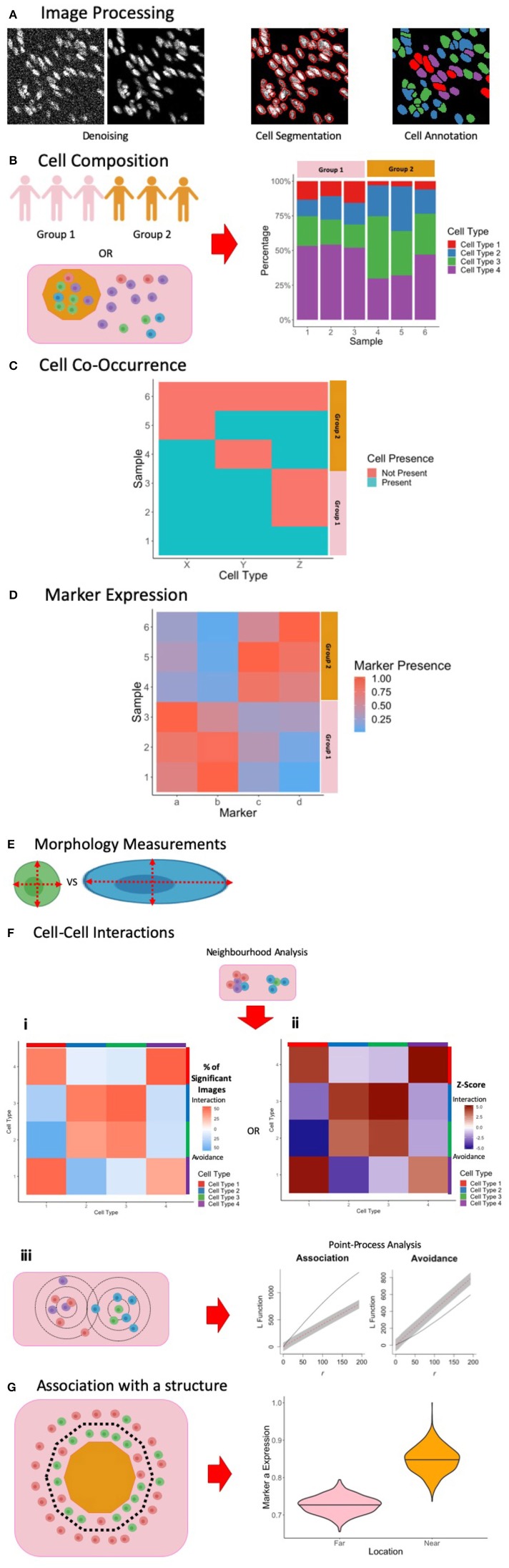
Summary of Image Processing and Analysis Techniques in MCI. **(A)** Following the acquisition of MCI, image processing is performed to denoise the images, perform single-cell segmentation to identify cell outlines, and to classify these cells based on marker expression. **(B)** One way of exploring cell composition between groups is to compare the change in the cell fractions. **(C)** Another way to explore cell composition is to classify patients as being positive and negative for a particular cell population. The co-occurrence of cells can be presented similar to what is presented here, and significance of co-occurrence can be identified using a chi-square test. **(D)** Differences in marker expression between patients can be visualized using a heatmap. **(E)** Cell morphology measurements can be used to explore cell phenotypes. **(F)** Cell-cell interactions can be measured using neighborhood analysis or point-process analysis. With a neighborhood analysis, percentage of significant images (i) or Z-scores (ii) of the cell-cell interactions can be represented as a heatmap, with significant associations associated with a more positive Z-score and significant avoidance is associated with a more negative Z-score. With a point-process analysis, an L function can be used to assess the significance of cell-cell interactions. The L function being above or below the gray envelope generated by bootstrapping corresponds to association and avoidance, respectively (iii). **(G)** One way of measuring cell or marker association with a marker is to classify cells as being near or far away from the border. A cell composition analysis can be used to explore differences, or differences in marker expression can be explored, as shown here. Parts of this figure were made Biorender.

An important issue common to all image analysis is the presence of noise and artifacts which must be removed prior to analysis (Figure 3A, left). Robust and stable methods for denoising will become increasingly important if MCI is to be applied within the clinical setting, allowing for accurate patient sample characterization. There are noise profiles that are specific to MCI, in contrast to other imaging technologies, and may be specific to tissue types (13, 17, 18, 102). To comprehensively analyse the images obtained, various computational methods for denoising to preserve real signal and remove technical artifacts have been proposed (13, 17, 18, 102). At present there is no consensus on the most appropriate way to denoise images with research to date employing homebrew approaches based on the level and composition of noise observed by the investigators. Such approaches include correcting for channel cross-talk (18, 102), removing objects that differ from real signals in terms of size and pixel distributions (13, 18), and by using image filters to identify artifacts (13, 18). Here, we describe methods proposed to eliminate noise and artifacts in MCI images.

Crosstalk is the phenomenon in which signals from one channel are introduced into adjacent channels. This has been observed when comparing channels within ± 3 atomic masses from each other, occurring due to the presence of contaminating isotopes of similar masses (103–105). Crosstalk can even occur within 16 atomic masses due to oxidation (105). This confounding phenomenon needs to be corrected as it can lead to the misidentification of real signal within a single channel, particularly if adjacent channels correspond to markers that may be co-expressed. To correct for crosstalk, two methods have been proposed. Wang et al. observed a linearly correlated increase in pixel intensities at high pixel values for adjacent channels when plotting the intensities for two channels (18). They classified these pixels as crosstalk, and compensation was performed by resampling their intensity values in the given channel, providing a post-acquisition method for correcting for crosstalk. Chevrier et al. presents a bead-based compensation workflow to account for crosstalk, made available as the CATALYST R/Bioconductor package (102). Damond et al. implements this solution, measuring channel crosstalk using a slide with the metal isotopes used. There are trade-offs between using a fully post-acquisition approach, as opposed to a bead-guided approach. The post-acquisition method by Wang et al. is advantageous as it minimizes IMC acquisition time and resources required. However, it is unclear if their approach is valid for other images, and it is difficult to assess if real signals are removed. Additionally, correction may not be necessary if the marker panel employed is well-designed and titrated. If certain markers are expressed at vastly different levels across samples, panel design alone may not eliminate crosstalk. Hence, users should make a judgement as to whether crosstalk correction is necessary for their study.

Background noise and the corrections required can be specific to certain tissue types and experimental setups. As such, several “homebrew” computational methods have been developed to identify and remove noise in MCI images. Wang et al. observed horizontal streak artifacts within their image (18). The authors accounted for this by using a 5 × 5 μm^2^ median filter which excludes the middle row. For each pixel, the median pixel value within this filter is measured, and the central pixel is removed if it is brighter than this median and is in the top 2% of pixel intensity values, characteristic of these streaks. Keren et al. observed a background artifact in areas of the slide outside of tissue in all channels (13). To correct for this, a background channel, not containing antibody derived signals, was obtained. The area corresponding to background was identified with a threshold, and the pixel intensities were reduced in all other channels within this area. The authors also observed that low density pixel signals (those with few neighboring pixel signals) were associated with noise, while real signals tended to aggregate together, corresponding to cellular staining. To remove the interfering low density pixels, each pixel in the image was assigned a score by calculating the average distance to the 25 nearest positive counts. A bimodal distribution was obtained, and pixels above the crossing point of the distributions were removed, corresponding to the low-density noise. This background removal method complements wet-lab based optimizations of blocking methods and antibody concentrations. Indeed, it can be very challenging to address all signal-to-noise issues for large antibody panels. As such, computational methods, as described here, are an important preprocessing step to ensure reliable downstream analysis.

While various custom algorithms have been successful for denoising in previous studies, the application of MCI within a clinical setting will require an improved understanding of the sources of noise for specific samples. The standardization of “best practice” procedures for sample processing, staining, and acquisition in addition to image post-processing methods will be necessary going forward.

#### Cell Segmentation

Fundamental to the study of tissues in health and disease is the identification and characterization of individual cells. In microscopy this is achieved through single-cell segmentation, which involves identifying the boundary of individual cells (Figure 3A, middle). While single-cell segmentation has been applied to higher resolution fluorescence images, MCI images present a unique challenge because of its lower resolution, making the identification of cell boundaries more difficult both visually and computationally. Accordingly, this section discusses several approaches which have been applied for segmenting MCI data (summarized in Table 3).

**Table 3:** Software for cell segmentation and cell classification.

	**Technique**	**Description**	**References**
Cell Segmentation	CellProfiler	Identify primary object with nuclear marker, secondary object with membrane marker	(110)
	Weighted sum of membrane markers	Segments using a weighted sum of membrane markers	(111)
	Ilastik	Uses a random forest classifier, defining pixels as nuclear, cytoplasm, and background based on user training data. Probability maps can be used as an input for segmentation in CellProfiler	(112, 113)
	DeepCell	Identifies cell nuclei based on training data, using deep-learning	(114)
Cell Classification	Manual gating	Users manually identify their cells based on marker expression	
	Hierarchical Clustering	Identifies clusters in a hierarchical cluster by grouping together cells or clusters that are most similar to each other	
	Phenograph	Models cells as a nearest-neigh graph in high-dimensional space	(115)
	FlowSOM	Self-organizing maps used to identify cell populations. Meta-clustering is then performed to find a given number of populations	(116)
	Ilastik	Uses a trained random forest classifier to classify identified single cells	(112, 113)

Pipelines for single-cell segmentation established for other imaging modalities are also popular for MCI. These pipelines typically apply a threshold to a nuclear image and implement watershed segmentation to identify nuclear boundaries. Dilation of the cells, or the use of a cell-membrane marker, identifies the remaining cell body. The popular CellProfiler software (53) is often used for single-cell segmentation, with the user being able to provide inputs on the size filters, smoothing, and thresholding applied among other parameters to achieve segmentation. This is implemented by Wang et al., taking advantage of the many parameters used in IMC by using a range of non-immune and immune cell membrane markers for cell segmentation (18). This approach has the advantage of not requiring user training, requiring few user inputs for implementation. However, CellProfiler may not be able to segment cells that are packed tightly, as in tumors and lymphoid tissues, especially when the resolution is low as in the case of MCI. Schüffler et al. proposes a method in which multiple membrane proteins are weighted together to define the cell membrane (106). The proposed method performs an exhaustive search for an appropriate weighting and smoothing of all cell membrane channels, and provides a score based on how successful segmentation is performed. This self-reflective scoring may be useful for assessing the success of segmentation, but it is unknown whether it is successful for difficult, high-density images. Finally, Durand et al. employs an in-house-developed segmentation pipeline to achieve single-cell segmentation of tonsil tissue (56). First, a Laplacian-of-Gaussian filter is applied, which resolves nuclei as spots with a local minimum. A h-minima transform is then applied to identify these local minima (115). Finally, a single-cell segmentation mask is obtained by applying a watershed transformation to the linear combination distance map obtained from the h-minima transform and the average image of all membrane-bound marker proteins. The cellular regions are restricted by a defined radius of 8 pixels around each local minimum to avoid oversized cells. Ultimately, these pipelines allow cell boundaries to be identified without user training.

For more precise single-cell segmentation in MCI, supervised classifiers have been successful. These approaches require humans outlining single-cells to produce a set of well-annotated cells that can be used to train machine learning algorithms, with the advantage that humans may be better at identifying the subtle details that separate cells. Schulz et al. (16) and Damond et al. (17) implement the popular Ilastik toolkit (112, 113), employing a random forest classifier for cell segmentation, while Keren et al. (13) implements DeepCell (116), which employs deep-learning for cell segmentation. With both tools, training sets are developed using nuclear, cytoplasmic, and membrane markers, and a probability map is produced describing whether a pixel is nuclear, cytoplasmic, or background. CellProfiler, or conventional thresholding and watershed segmentation is then used to identify cells and their bodies based on the probability maps. This workflow of performing segmentation on probability maps was first demonstrated by Schapiro et al. (53). These supervised methods have been successful at separating cells that are clustered together, and can be advantageous to using CellProfiler in a standalone manner. However, these techniques require users to generate substantial training data with a new classifier needing to be generated for each experimental panel and tissue type which can be time consuming.

In general, if the outline of cells is obvious, using CellProfiler may be sufficient for performing single-cell segmentation. However, if cell shapes are more complex, as in the case of neural tissue, or if dense cell structures are present within tissue structures, then the use of classifiers will be more suitable. In fluorescence images, these classifiers have been shown to outperform classical methods for segmentation (117), but an extensive comparison using MCI has not yet been performed. As the use of MCI becomes more universal and applied within a clinical setting, there will be an increased need for more precise segmentation. It is likely that the most appropriate method will be to use a well-trained classifier. For generally applicable classifiers, users may have to contribute to an existing online classifier, creating a diverse training set to perform cell segmentation. Much investigation will hence be necessary in the future for improved and more generalized segmentation.

#### Tissue and Cell Annotation

Immune cells exist in great diversity within both healthy and diseased contexts. Along with canonical cell types such as Dendritic Cells, Macrophages, T cells, and B cells, each cell type is comprised of diverse subsets which differ throughout the body. Importantly, specific subsets can play a crucial role in disease manifestation, even when their prevalence is extremely low. As such, accurate and high throughput methods for the annotation of cell types (Figure 3A, right) and the tissue compartments in which they reside, are essential. Here we discuss several approaches that have been employed for the annotation of MCI data (summarized in Table 2).

The simplest approach for identifying cells is by selecting manual gates based on scatter plots of marker expression, similar to other single-cell technologies such as flow cytometry. Marker expression is typically quantified by summing the ion counts within a single cell as outlined by segmentation and dividing by the area of the cell. The histoCAT package (53) provides a tool which allows users to gate on cells and visualize the presence of these cells within their image. Furthermore, the single cell data can be exported from histoCAT for downstream analysis using commercial platforms such as FlowJo or Cytobank, and also open source platforms such as Flowing Software. However, a key advantage of histoCAT is that cell selections can be visualized on the image in real-time, which facilitates greater accuracy when selecting gates. However, while manual gating provides a user with full control over the cells being classified, this can be time consuming, especially when many markers are considered. Nevertheless, manual gating may be useful for exploratory analysis of image data.

One approach for semi-automated gating is by using a mixture model, such as the implementation by the *mclust* R package (106). This package is used to classify cells as being positive or negative for a marker, based on the mean pixel intensity in that specific marker channel. Another approach is by Boolean rules based on whether cells are positive or negative for these markers to classify cell types. Wang et al. implement this method, but set additional manual cutoffs as informed by the mixture models to identify positive and negative populations (18). This approach will only be applicable for markers with which cells can be discretely positive and negative for, but not when cell-type definition relies on a continuum of marker expression (e.g., low, mid, and high).

Automated gating strategies employing clustering techniques to group cells by similarities in marker intensity have become popular in all high-parameter imaging assays. This provides a quick and unbiased approach for classifying cells in tissue. Schulz et al. (16) employs PhenoGraph (115) to cluster cells, employing a nearest-neighbor graph to identify phenotypically coherent subpopulations. Here, they use both marker expression as well as RNA expression to cluster cells. Durand et al. use a hierarchical clustering approach on all markers, obtaining 60 clusters which was arbitrarily chosen to overclassify cells (56). This allowed the authors to identify smaller yet distinct clusters with some similar clusters manually merged when the clusters were annotated based on known cellular phenotypes. Keren et al. (13) clusters cells into immune and non-immune cells using FlowSOM (116), which employs a self-organizing map to identify cell populations. Lineage marker expression was used to cluster cells. This was applied iteratively, first to distinguish between immune and non-immune cells, then to classify non-immune cells into epithelial, mesenchymal, endothelial, and unidentified cells, and finally to classify immune cells into specific subsets. The approach taken by Keren et al. employs only canonical cell markers, leading to the identification of canonical cell subsets. Expression of functionally significant markers was then assessed on the defined cell subsets in different tissue compartments. In contrast, Schulz et al. clusters using all markers, leading to canonical cells being divided by marker expression. For example, two CD3 high T cell clusters were obtained, one of which expressed CD3 only, and the other being a potential memory T cell subset. Importantly, this clustering revealed the identification of rare cells that express CXCL10 RNA. Durand et al., however, merges clusters with a similar phenotype. Hence, a choice needs to be made as to whether to include all markers or only lineage markers when investigating cell phenotypes.

Finally, users can employ supervised classifiers, providing training data to predict cell types based on both marker expression and the visual texture of the signals. For example, membrane markers will be localized only to the membrane of the cell. This can be achieved using an interactive classifier such as Ilastik, where users can annotate cells as the cell subsets they are interested in Ilastik uses both marker expression level and morphology to classify cells based on the provided training data. Damond et al. implements this classification iteratively, first to classify cells as islet, immune, exocrine, and “other” cells. A second round of training and classification was then performed to classify the different immune, islet, and exocrine cells, and “other” cells were classified as endothelial, stromal or unknown cells. The classifier is advantageous as classification is informed by both marker expression and texture as defined based on more reliable human judgement. However, the training of a classifier can be time consuming, and this approach will only be able to identify user-defined cells. Hence, supervised classifiers will not identify other cell marker phenotypes that automated gating may identify.

Following cell classification, tissue compartment identification can be performed. This is useful for exploring the role of tissue structures in the context of disease. Keren et al. (13) and Wang et al. (18) use classified tumor cells and islet cells to identify the tumor and islet areas, respectively. Damond et al. uses Ilastik to identify islets and blood vessels by constructing training data using a range of structural markers, while Durand et al. uses E-Cadherin, CD19, and CD3 to identify the crypt, B cell zone, and T cell zone of tonsil tissue. The identification of these tissue structures is important because of their role in disease pathology. For example, the tumor-immune boundary has been used as a prognostic indicator for tumor progression, and islet cell composition and morphology have been observed to change with disease progression (118–120). Identifying these key compartments and their borders hence allow their role to be observed with MCI.

Similar to the segmentation of individual cells, cell-type annotation in the clinical setting would require automated and standardized methods for cell-type classification. At present, classifiers used for annotation are trained on a study-by-study basis. Although accurate, it has not been established that these approaches are generalizable or time efficient for use in the clinical setting. Ultimately, classifiers will need to be constructed and trained to account for patient and experimental variation.

### Analysis

In this section, the key biological questions that are answered through image analysis is discussed (summarized in Table 4).

**Table 4:** Summary of analytical questions with clinical examples and the techniques used to answer these questions.

**Analytical question**	**Clinical example**	**Analytical techniques**
How does cell composition change with disease context?	How does cell composition change with type-1 diabetes progression? (17, 18)	Measurements such as cell counts, cell proportions, or cell densities can be used to compare between different disease contexts Pearson's correlation of the above measurements can be used to identify the co-occurrence or anti-occurrence of cell types Cell types can be considered present or not present within an image if the cell count is greater than a given cut-off (e.g., 10 cells). A chi-square test can then be used to identify cell type co-occurrence
Does marker expression or co-expression change with diseased context?	How does islet marker expression change with type 1 disease progression? (17)	Heatmaps can be utilized for visualizing marker changes across images Markers can be considered present or not present within an image. A chi-square test can then be used to identify marker co-occurrence Pseudotime analysis such as SCORPIUS (121, 122) allow marker changes associated with cell dynamic processes to be investigated
Does cell or structural morphology change with diseased context?	Does islet morphology change with disease progression? (17)	Morphology measurements can be identified using image analysis software such as histoCAT (53), CellProfiler (110), and ImageJ (123)
Are there any interactions between specific cell types, and does this change with disease context?	Are tumor-immune interactions present and significant within tissue compared to immune-immune interactions? (13, 53)	Neighborhood analysis using histoCAT (53), or by setting a distance cut-off to define neighbors (13), can be used to identify cell interaction or avoidance, visualized with a heatmap Marked point process models using the R package “*spatstat”* can be used to determine cell co-localization or anti-co-localization (124, 125)
Do cells localize to histological structures and does this vary with disease context?	In breast cancer sections that exhibit compartmentalized structures, are there differences in marker expression with distance from the tumor-immune boundary? (13)	Within binned distances away from a histological boundary, differences in cell composition (17, 18) or marker expression (13) can be identified Marked point process models using the R package “*spatstat”* can be used to explore the distribution of cells as a function of distance from a histological boundary (124, 125)
What is the role of the cell microenvironment in a diseased setting?	In multiple sclerosis brain lesions, how does the environment influence variations in cell marker expression? (55)	Spatial variance component analysis (126) can be used to decompose the sources of variation of a marker into intrinsic effects, environmental effects, and cell-cell interactions

#### How to Stratify Data for Analysis?

To understand the biological processes underlying disease, the appropriate stratification of patient data for analysis is important. The simplest method is to group data based on clinically defined categories such as “time since diagnosis” or “patient survival.” This approach is implemented by both Wang et al. (18) and Damond et al. (17) in their study of T1DM. Here, they stratified their patient groups based on time since diagnosis, with an additional control group. Although this method is often appropriate, stratification based on a biologically meaningful model of disease can offer a powerful and complementary approach for revealing disease specific relationships that simple clinical groupings could miss. For example, as diabetes is a progressive disease, Damond et al. performed pseudotime analysis (discussed below) to group islets into three “pseudostages” of disease. This followed from their observation that islet profiles followed a spectrum during the early-stages of disease, resembling both healthy islets and late-stage islets as well-intermediate stages in between. Additionally, as tumor-immune organization is known to predict survival for certain cancers, Keren et al. performed a spatial enrichment analysis (discussed below), generating a metric for tumor-immune cell mixing and allowing the investigators to stratify patients based on tumor organization (13). The decision on how to best group data for analysis is crucial for the discovery of disease specific immunological motifs. In reality, this part of the analysis stretches back to experimental design. To effectively use MCI as a primary research tool, it is important to carefully consider beforehand, choice of patient samples, availability of clinical data and also MCI panel design. These three aspects will inform the types of data stratification that are possible and therefore the scope of questions that can be asked and answered using MCI.

#### How Does Cell Composition Change With the Disease Context?

The prevalence of specific cell subsets is associated with disease outcomes, both in the clinical setting and in models of disease. As such, the basic analysis of cell composition is an important first step which can also inform downstream analyses. In present MCI studies, this has taken two approaches. The first is to quantify cell compositions and then compare these between different patient groups. This is done as either absolute counts of a specific cell subset, a measure of its proportion among a larger group of cells, or as a cell density per mm^2^ of tissue. The second is to examine the co-occurrence or anti-occurrence of cell types, providing an insight on any causal pathways that may underlie disease. In this section we summarize how MCI studies have explored cell composition within tissue.

In MCI studies, the cell subset composition can be presented as the proportion of the total cells (or all immune, tumor, islet cells, etc.) (Figure 3B), the total number of cells, or the cell density. There are many advantages and disadvantages to these different approaches for quantification. Total counts can allow for patient-patient comparisons, allowing interpatient variations to be observed. When comparing between groups of patients, the cell proportion may be more appropriate for comparison, normalizing the data to account for interpatient variation. Cell density per mm^2^ of tissue may be appropriate when comparing cells within compartments, with the data being normalized by the area of the compartment. The density measurement is also useful for comparing small changes that are overwhelmed by the abundance of another cell type. Ultimately, the choice of measurements used is dependent on the question being asked.

This cell composition analysis is implemented by both Damond et al. (17) and Wang et al. (18) in studying how the islet cell composition changes with T1DM progression. Both studies observed a decrease in beta cell fraction, and an increase in gamma cell fraction with disease progression, relative to all other islet cells. Damond et al. further observed a small decrease between pseudostage 1 and 2 islets, followed by a significant decrease between pseudostage 2 and 3 islets. Additionally, Damond et al. and Keren et al. present the proportion of immune cell subsets within their images, assessing the composition of immune infiltration within tissue. Data obtained from cell composition analysis can also reveal meaningful biological relationships. For example, Keren et al. ordered patients by number of infiltrating immune cells and found that patients with more immune cells were more likely to have a “compartmentalized” phenotype. Additionally, Damond et al. found that when ordering patients by the number of islet cells, stratified by patient diabetes status, mid-sized islets had a higher proportion of beta cells. Presentation of data in this manner can aid in the interpretation of single-cell MCI data.

To assess cell subset co-occurrence or anti-occurrences, two approaches have been used in present MCI studies. The first approach is to observe whether the count or proportion of one cell subset is correlated with that of another cell subset, assessed using Pearson's Correlation. This measurement is useful when investigators want to show that an increased presence of one cell type is accompanied by an increase or decrease of another cell type, and is appropriate when both cell types are often or always present within that tissue type. The second approach is to convert cell counts into categorical data by classifying images as being positive or negative for a given cell subset if the count exceeds a user-defined cutoff (Figure 3C). A chi-square test is then used to quantify the significance of co-occurrence. This measurement is not very useful when both cell types are often or always present within that tissue type. Hence, this measurement is suitable only when the cell types being investigated are not consistently present within that tissue type.

The co-occurrence approaches mentioned above have been applied by Keren et al. and Damond et al. In studying immune infiltration into tumors, Keren et al. observed that there was a correlated increase in CD4+ T cell proportion and a correlated decrease in macrophage proportion. Similarly, when studying immune cell infiltration into the islets, Damond et al. observed a correlated increase in CD4+ helper, and CD8+ cytotoxic T cells in pseudostage 2 islets. This revealed that both CD4+ and CD8+ T cells are recruited simultaneously into the islets during the onset of diabetes, potentially co-operating to mediate beta cell destruction. Furthermore, to assess co-occurrence of cells in tumor infiltration, Keren et al. classified each patient as being positive for a given immune cell if the cell count is >10, and negative otherwise. A chi-square test subsequently revealed relationships such as patients with B cell infiltration into their tumors also had CD4+ and CD8+ T cell infiltration. The relationships observed by these analyses reveal a potential coordination in the immune response in both tumors and islets, with the recruitment of several cell types occurring.

#### Does the Expression or Co-expression of Cell Markers Change With Disease Context?

In addition to changes in cell composition, understanding variations in functionally relevant markers is essential for understanding disease pathology. Indeed, many interventions targeting cancer, infectious diseases and autoimmune diseases use antibodies and small molecule inhibitors targeting cytokines or cell-associated ligands/receptors (127–129). Through the many markers afforded by MCI, these diverse markers can be studied within the disease pathology setting. This section will explore how marker expression is examined in IMC images.

In MCI studies so far, the exploration of cell marker expression has taken many pathways. One approach is to compare marker expression among canonical cell subsets, with fold-changes being expressed as a heatmap (Figure 3D). Marker expression can also be measured at the tissue compartment or patient level, with expression level visualized as a heatmap for each sample (Figure 3D). By stratifying samples into groups, direct comparisons can be made. To assist with the analysis of the many markers used by MCI imaging, dimensionality reduction techniques have been used. These include principal components analysis, t-Distributed Stochastic Neighbor Embedding (t-SNE), and pseudotime analysis. Finally, the investigation of preferential co-expression of markers can be assessed by classifying images as being positive or negative for a given marker and using a chi-square test to quantify the significance of co-occurrence. Each of these approaches can be used to investigate differences in marker expression within different samples, with each analysis telling different aspects of the overall pathophysiological story. Investigators should use the appropriate investigation required depending on the question being asked, and the cellular pathway being explored.

In their investigation of T1DM progression, Damond et al. studied the change of islet marker expression within islets (17). While the investigators observed a decrease in beta cell fraction as described previously (120), they wanted to further investigate whether this was a result of beta cell loss, a downregulation of beta cell marker expression, or both. To investigate this, the authors performed a pseudotime analysis using the trajectory inference algorithm SCORPIUS (121, 122). This was performed by measuring the islet marker expression profiles of each individual islet. The algorithm finally assigns a value between 0 and 1 to each islet, relating the marker expression profile of islets to the T1DM development timeline, and allowing the investigators to stratify the islets into three pseudostages. Specifically, they observed a strong downregulation of beta cell markers between pseudostages 1 and 2, and stability between pseudostages 2 and 3. The authors concluded that progression from pseudostages 1 and 2 may be driven by the down regulation of beta cell markers, while the transition between pseudostages 2 and 3 is reflective of cell death. The assessment of changes in marker expression, combined with cell composition analysis, can reveal the mechanisms behind a disease timeline.

Keren et al. investigated the expression of the immunoregulatory proteins PD-1, PD-L1, IDO, and LAG3 in their study of breast cancer (13). Through a chi-square test, they found that patients expressing one of these proteins expressed another, implying that multiple immunosuppressive pathways are present within the tumor environment. Additionally, it was found that the presence of regulatory T cells accompanied the presence of at least one of these markers, reflecting the potential for these proteins to induce the differentiation of naïve T cells toward a regulatory T cell phenotype. Such results provide insight as to the signaling pathways that are present within the disease setting, and relate molecular expression profiles to the histological structure of the tissue.

Ultimately, it is important to understand the distribution and expression level of functional markers relevant to disease. These maybe chosen based on the literature as in the MCI studies discussed here, or alternatively using other omics technologies, such as genomics and proteomics platforms (130, 131), to pre-screen samples for suitable candidates. Importantly, the inclusion of such markers allows one to infer biologically processes from static 2D images.

#### Does Cell or Structural Morphology Vary With Disease Context?

Another important aspect of cellular phenotype is its morphology (Figure 3E). Just as with marker expression, cell morphology can also be associated with disease context or with drug treatment. Morphology measurements such as area, perimeter, solidity, eccentricity, and circularity can be made with analysis software such as histoCAT (53), as well as most image analysis packages (110, 123). These measurements allow structural changes to cell or tissue to be identified with changing disease context, or with drug treatment. However, the reliability of the measurements is dependent on how accurately segmentation of objects are obtained. This can be difficult with the lower resolution of MCI images, but may be reliable when classifiers are used, as mentioned previously.

Morphological measurements can be used to assess the integrity of histological structures. Damond et al. applies these measurements to their islets to assess changes with diabetes progression (17). The authors measured the islet extent (islet area divided by islet bounding box) and solidity (portion of pixels in the islet convex hull that are also in the islet), indicative of shape regularity. These two measurements were found to decrease between pseudostages 2 and 3, indicative of a more irregular islet shape, associated with beta cell loss and diabetes progression. Thus, morphology measurements can provide an unbiased quantification of tissue structure, identifying degradation as described here, but may also be used to highlight swelling or growth.

Cell morphology can be affected by drugs and has utility in drug-discovery (132). Bouzekri et al. uses morphology measurements to assess drug effects on breast cancer cell lines as visualized by IMC. The authors found that certain drugs led to an increase in size, with morphological measurements such as area, perimeter, and major- and minor-axes increasing following drug application. In combination with protein measurements, these observations may allow researchers to propose transduction pathways affected in response to drug treatment (133, 134).

#### Are There Any Interactions Between Specific Cell Types Within Tissue, and Does This Change With Disease Context?

Within previous MCI studies, two methods have been used to investigate cell-cell interactions. The first is through the neighborhood analysis algorithm described by Schapiro et al. (53). This method identifies whether a cell of type X is within a user-defined neighborhood of cell type Y, and vice-versa. This is performed by dilating each cell in a single-cell mask by a user-defined number of pixels (usually 4–6) and counting the cell types that it overlaps with. To assess significance, a bootstrapping approach was implemented, in which the annotated cell labels are randomly reassigned. The mean number of cells of type X within the neighborhood of cells of type Y are calculated for each simulation and for the real distribution. The statistic obtained for the real distribution is then ranked against the simulated statistics with two one-tailed permutation tests to obtain a *p*-value. The upper-tailed test corresponds to interaction, while the lower-tailed test corresponds to avoidance. When applied to a large number of donors, this can be represented on a heatmap as the percentage of significant avoidance or interactions for each cell pair (Figure 3Fi). The second method was to count the number of cells of type X within a user-set distance away from cells of type Y. A similar bootstrapping approach was implemented, and the number of cells was remeasured to generate a distribution from which Z-scores are obtained. This relabeling can be performed with all cells, providing context of global organizational patterns of the cells, or by constraining within a specific group of cells (e.g., immune cells, tumor cells, T cells, etc.), providing a more context dependent answer. A negative Z-score corresponds to avoidance, while a positive Z-score corresponds to association, and these values can be visualized on a heatmap (Figure 3Fii). These two approaches are effective for identifying cell-cell interactions. However, they do not provide any context of the cell-cell interactions over a wider distance, and does not reveal whether cells traffick toward a particular target. Additionally, an arbitrary distance needs to be chosen, and the sign of the Z-score and hence the interpretation of cell-cell interactions, can vary with scale.

This neighborhood analysis technique has been applied by Damond et al., who observed reduced beta cell associations in the third pseudostage, representative of beta cell destruction, while immune cell associations with other immune cells was increased in the second and third pseudostages, indicative of an immune response (17). They also found that the number of interactions of beta cells with CD4+ helper and CD8+ cytotoxic T cells was much higher during pseudostage 2, in line with their previous results.

Additionally, Keren et al. counted the number of cells positive for marker X located within 39 μm from marker Y. This resulted in the identification of three distinct levels of tumor and immune cell mixing: “cold,” with low immune infiltration, “mixed,” with high immune infiltration, and “compartmentalized,” with tumor and immune cells forming distinct clusters separated from each other. The authors developed a mixing score to quantify this, defined as the number of immune-tumor interactions divided by the number of immune-immune interactions. Furthermore, when plotting Kaplan-Meier curves, which showed survival as a function of time for patients, they observed higher survivability in patients with “compartmentalized” tumors compared to patients with “mixed” tumors. Here, the spatial organization of tumor was related to patient survivability.

To explore avoidance or association at a range of distances, cells can be modeled as a marked point process model (135), in which cells are represented as labeled points on a plane. One approach is to use Ripley's K and L functions to model cell-cell interactions, with the variance stabilized L function being a useful transformation to the K function (plotted in Figure 3Fiii). Simply, Ripley's K function is a function which models the number of cells of type X a certain distance away from cells of type Y, as a function of distance. Bootstrapping is once again used to generate significance. This was used by Setiadi et al. in fluorescence imaging to show that B cells cluster in tumor-draining lymph nodes compared to healthy lymph nodes (136). While applied to the same cell type in this example, this can be applied to pairs of cells of different types, or with cells of a specific type to a pathogen. This can provide context of the significance of these interactions along a wider range, and to observe how interactions can change with scale, and may give insight to any cellular trafficking from a steady-state image. These functions, along with other functions and methods for comparison between samples, are readily available in the R package *spatstat* (124). However, a disadvantage of these models is that no single Z-score is given, making visualization and interpretation difficult. Baddeley et al. (125) proposes envelope-based tests to measure the statistical power of the interaction or avoidance, while another strategy may be to determine the percentage of images with which interaction or avoidance was significant, similar to Schapiro et al. (53). An investigation on the appropriate spatial statistic will be necessary to make robust conclusions about any cell-cell interactions, especially in the context of MCI images where many cell subsets are being investigated simultaneously.

With MCI, spatial analysis can be applied to a wider range of cell subsets compared to conventional microscopy over a range of distances. This allows a diverse range of cell-cell interactions to be performed, with the possibility of cell-pathogen interactions to be investigated in the future. Although these images only provide a snapshot of the tissue environment, the identification of significant interactions may bypass the need for more complicated techniques using live imaging. As well, interactions observed in the native microenvironment provide a sound rationale for *ex vivo* co-culture experiments, to investigate the functional outcome of certain cell-cell interactions. Given that specific cell-cell interactions have already been associated with patient outcomes (13, 17), such interactions metrics could prove a useful prognostic indicator in a variety of disease settings.

#### Do Cells Localize to Histological Structures and Does This Vary With Disease Context?

In addition to cell-cell or cell-pathogen interactions, it is useful to understand whether cells or pathogens localize to a specific histological structure, such as epithelium, tumors, and islets, which have been implicated to have an involvement in disease pathology.

In previous MCI studies so far, the number of cells or the amount of cell expression was measured within user-selected binned distances from the structure to investigate cell localization. This can be visualized as a heatmap, if 2 bins are used to represent “near” or “far” from a border. Dividing distance from a structure into user-selected bins essentially turns the problem into a comparison of cell composition or marker expression between bins. While simple, this approach discretizes continuous data, and results can vary depending on the bins used. In particular, it is hard to ascertain whether there truly is a continuous trend in the change in cell composition or marker expression toward a border. The use of a point process model as described in the previous section may prove to be suitable for analyzing the spatial dependence of cells or markers from a structure, but further investigation is required to assess the robustness of such measurements.

Keren et al. hypothesized that there are differences in the cell phenotype near or far from the tumor-immune border in “compartmentalized” patients (13). To investigate this, the authors applied a cut-off of 39 um to stratify cells as being close to or far from the border. In addition to counting cells, they observed whether marker expression was higher or lower away from the border (Figure 3G). The authors observed that the ratio of H3K27me3 (methylated DNA) to H3K9ac (acetylated DNA) increased for tumor cells that are far from the border in two patients, indicating that cells closer to the border may be more transcriptionally active. However, this strategy fails to provide any insight as to how this marker ratio varied continuously as a function of distance from the border. It would be interesting to see whether or not the marker ratio increased with distance, coinciding with the binned approach, or whether it alternates between increasing and decreasing. Furthermore, to simplify the spatial relationships observed, a principal component analysis was performed, revealing a subset of patients that had increased immunoregulatory protein expression in CD11c+CD11b+ immune cells. This is suggestive of myeloid derived suppressor cells, which may inhibit the immune response (137). Hence, the examination of a spatial binning to analyse the spatial dependence of marker expression from a structure was able to reveal subgroups of patients with unique phenotypes. An interesting progression may be to compare how the survival varies between these subgroups.

#### What Is the Role of the Cell Microenvironment in a Diseased Setting?

Multiparameter imaging provides the opportunity for cellular microenvironments to be examined within a diseased setting. Spatial variance component analysis (SVCA) (126) is a technique that has been applied to MCI data which allows the sources of variation of gene or protein markers in an image to be identified, without the need for cell classification. The sources of variation of cell markers are decomposed into intrinsic effects, environmental effects, and cell-cell interactions. SVCA was applied by Park et al. to investigate how multiple sclerosis (MS) brain lesion environments influence variations in cell marker expression (55). They found that toward the center of a lesion, the relative influence of intrinsic and environmental effects increased, while the relative influence of cell-cell interactions had decreased. The authors suggest that cells in the lesion rim are more responsive to cues from the microenvironment, such as cytokines or receptor-ligand interactions, while cells respond to cell-intrinsic programs in the lesion center. There have been additional methods proposed for measuring associations between cell microenvironment and marker expression, however these methods have not been applied to MCI data (7, 138). Ultimately such analysis approaches can provide insight on the role of the microenvironment within a diseased state.

### Summary of Image Processing and Analysis

Through image processing and analysis, researchers are in a position to interrogate high parameter MCI data in a single-cell manner. This approach allows key clinical and biological questions to be explored and answered, providing insight on the cellular dynamics that are present in the diseased context. In addition, these results can inform further experimentation within or outside the cytometry setting.

There is potential for the development of statistical tests to identify associations between disease outcomes and the spatial relationships between cells, implementing spatial information with multiple markers. Current methods are able to classify cells, but still perform simple spatial analysis that is implemented in other imaging cytometry assays (135). Complex machine learning algorithms will eventually benefit from including both spatial and marker information provided by MCI, constructing predictive models in a higher dimensional space.

Deep learning has become a well-established tool for image analysis. Its consistent use in a variety of applications has been driven by its ability to deconstruct and model highly complex images (112, 113, 115, 116). However, deep learning methods require many observations to train effective models. In MCI, deep learning is ideal for cell type prediction, where thousands of cells can be trained from a single image. Though, it is unclear whether it will be effective for classifying heterogeneous global spatial interactions in datasets with relatively small sample sizes, as observed in many exploratory clinical studies. Such approaches may become useful in large cohorts generated after MCI has been implemented in routine clinical use, allowing for improved accuracy.

There is still an exciting opportunity to develop analytic algorithms for summarizing spatial cell-cell interaction relationships into simple, easy to interpret summary statistics. Such algorithms are characterized by the discussed methods for tumor-immune mixing quantification and pseudotime analysis, which stratify patients into risk groups or assigns groups to a disease progression gradient. It is important to simplify such complex relationships as it will allow scores or statistics to be developed for interpretable decision making. This may also facilitate the ability for MCI data to be included in disease risk scores, incorporating the data with other clinical and pathological and genetic information (13, 117–119).

Finally, to establish confidence in the use of MCI in the clinical setting, it will be important to identify methods that are both accurate and robust in a variety of applications. This focus applies to both methods for quantifying differences in cell-type compositions and spatial interactions, as well as strategies for image processing and quality control. Within the MCI analysis community, there has been an established culture of making code available on repositories such as github, as well as supplemental information when research is published, with image data also being openly accessible. It is important that this culture continues, supporting methods that are well-annotated, easily implemented, and actively maintained for reliable integration with multiple pipelines. This will ensure that robust and novel analytical development strengthens the potential for MCI, pushing the technology closer toward clinical application.

## Conclusions

In this review we have outlined the various applications of Mass Cytometry Imaging for studying the immune system in health and disease *in situ*. MCI is a more recent addition to the repertoire of tools for high parameter imaging. However, despite its recency it has already been adopted in diverse contexts ranging from oncology to autoimmunity where it has shown promise for predicting clinical outcome and understanding the role of the immune system in disease progression. Underlying these studies are common questions relating to the composition, phenotype and location of cell subsets and how they interact. Given the fundamental similarities, these studies also share similar computational strategies which we have linked to the general biological questions they answer. MCI is currently limited by its speed of acquisition, which often restricts analysis to smaller areas, and also the availability of commercially available pure isotopes. The speed has improved substantially since the initial papers describing IMC and MIBI, and will likely continue to improve as new advancements are made. At present, this limitation can be mitigated by using MCI with complementary assays such as immunofluorescence microscopy which can guide the selection of regions to be acquired by MCI. As advancements are made in instrumentation and reagent availability, computational tools, which are still in their infancy, must also develop to realize the full potential of high parameter image data. We anticipate MCI in combination with other high dimensional assays will play an important role in furthering our understanding of the etiology of disease and in clinical decision making.

## Author Contributions

HB, NC, AC, AH, and EP conceived and planned the manuscript. HB and NC performed the literature search. HB, NC, and EP drafted the manuscript. AC and AH provided critical revisions.

### Conflict of Interest

The authors declare that the research was conducted in the absence of any commercial or financial relationships that could be construed as a potential conflict of interest.
